# Identification of *Gossypium hirsutum* long non-coding RNAs (lncRNAs) under salt stress

**DOI:** 10.1186/s12870-018-1238-0

**Published:** 2018-01-25

**Authors:** Fenni Deng, Xiaopei Zhang, Wei Wang, Rui Yuan, Fafu Shen

**Affiliations:** State Key Laboratory of Crop Biology, College of Agronomy, Shandong Agricultural University, Tai’an, 271018 Shandong People’s Republic of China

**Keywords:** lncRNA, Salt stress, *Gossypium hirsutum*, RNA-Seq, RT-qPCR

## Abstract

**Background:**

Long non-coding RNAs (lncRNAs) represent a class of riboregulators that either directly act in long form or are processed into shorter microRNAs (miRNAs) and small interfering RNAs. Long noncoding RNAs (lncRNAs) are arbitrarily defined as RNA genes larger than 200 nt in length that have no apparent coding potential. lncRNAs have emerged as playing important roles in various biological regulatory processes and are expressed in a more tissue-specific manner than mRNA. Emerging evidence shows that lncRNAs participate in stress-responsive regulation.

**Results:**

In this study, in order to develop a comprehensive catalogue of lncRNAs in upland cotton under salt stress, we performed whole-transcriptome strand-specific RNA sequencing for three-leaf stage cotton seedlings treated with salt stress (S_NaCl) and controls (S_CK). In total we identified 1117 unique lncRNAs in this study and 44 differentially expressed RNAs were identified as potential non-coding RNAs. For the differentially expressed lncRNAs that were identified as intergenic lncRNAs (lincRNA), we analysed the gene ontology enrichment of *cis* targets and found that *cis* target protein-coding genes were mainly enriched in stress-related categories. Real-time quantitative PCR confirmed that all selected lincRNAs responsive to salt stress. We found lnc_388 was likely as regulator of Gh_A09G1182. And lnc_883 may participate in regulating tolerance to salt stress by modulating the expression of Gh_D03G0339 MS_channel. We then predicted the target mimics for miRNA in *Gossypium*. six miRNAs were identified, and the result of RT-qPCR with lncRNA and miRNA suggested that lnc_973 and lnc_253 may regulate the expression of ghr-miR399 and ghr-156e as a target mimic under salt stress.

**Conclusions:**

We identified 44 lincRNAs that were differentially expressed under salt stress. These lincRNAs may target protein-coding genes via *cis-*acting regulation. We also discovered that specifically-expressed lincRNAs under salt stress may act as endogenous target mimics for conserved miRNAs. These findings extend the current view on lincRNAs as ubiquitous regulators under stress stress.

**Electronic supplementary material:**

The online version of this article (10.1186/s12870-018-1238-0) contains supplementary material, which is available to authorized users.

## Background

Long noncoding RNAs (lncRNAs) are arbitrarily defined as RNA genes larger than 200 bp in length that have no apparent coding potential. lncRNAs have emerged as playing important roles in various biological regulatory processes and have more tissue-specific expression patterns than mRNA [1–4]. It is well known that lncRNA transcripts are transcribed by RNA polymerase II mainly. As polymerase II polyadenylated products, lncRNAs are modified with a 5′-cap and (or) polyadenylated at the 3′-end in animals and plants [5–7]. The transcription of several lncRNA genes requires specific transcription factors, mediator complexes, histone modification complexes and transcription elongation factor complexes [6, 8–11]. Based on their genomic origins and their location relative to nearby protein-coding genes, lncRNAs can be classified into five groups: (i) sense long non-coding RNA, (ii) natural antisense transcripts (NATs) derived from introns, (iii) long intergenic non-coding (nc) RNAs (lincRNAs), (iv) intronic ncRNAs (incRNAs) and (v) bidirectional long ncRNA [7].

With advances in sequencing and the emergence of new technologies, the discovery process of lncRNA has generally developed in three phases: in the 1980s–1990s, individual lncRNAs, for example, *XIST* and *H19*, were discovered through traditional gene mapping approaches. *XIST* has been identified in mammalian cells, has no significantly conserved open reading frames and does not appear to encode a protein. In addition, *XIST* originates from the X-chromosome inactivation centre and functions as a structural RNA in the nucleus [12]. In the early 2000s, the development of large-scale cDNA sequencing led to the discovery of a surprising number of lncRNA transcripts. Especially in plants, thousands of lncRNAs have been identified, such as in *Arabidopsis thaliana* [13], rice [14, 15] and *Medicago truncatula* [16]. During the mid-2000s, the number of predicted genes in the mammalian genome declined, while the number of detected lncRNA transcripts increased exponentially in several plant species. Microarrays, tiling arrays and next-generation sequencing were used as high-throughput tools for identifying new transcripts [7, 17–19].

In the nucleus, lncRNAs may execute their functions either in close proximity (*cis*-acting) or over a distance (*trans*-acting) in the genome via numerous mechanisms, including activating, gathering or transporting proteins and epigenetic silencing and repression, modifying promoter activities by nucleosome repositioning, epigenetic modification by regulating the level of DNA methylation and histone modifications [3, 17, 18]. Increasing evidence has shown that lncRNAs play a crucial role in growth and development, disease occurrence and genetic and epigenetic regulation in mammals [20, 21].

Recent studies have reported on the function of lncRNAs in plants [18, 19]. Boerner et al. (2012) identified 2473 lncRNAs in *Zea May*, classifying small RNA precursors and lncRNAs that are likely to function as longer molecules [22]. In *Arabidopsis*, *COOLAIR* and *COLDAIR* have been identified to regulate the expression of *FLOWERING LOCUS C* (*FLC*) [18]*.* A lncRNA that has been identified in *Arabidopsis,* Induced by phosphate starvation 1 *(IPS1),* can bind and sequester miR399 and reduce miR399-mediated cleavage of Phosphate 2 (PHO2), which is associated with phosphate uptake [23]. In hybrid rice, long-day–specific male-fertility–associated RNA (*LDMAR*) has been found to regulate photoperiod-sensitive male sterility (PSMS) [24]. More recently, more than 2000 lncRNAs were identified in rice during sexual reproduction, including 1624 lincRNAs and 600 long noncoding natural antisense transcripts (lncNATs) [25].

Cotton (*Gossypium hirsutum L.*) is an important economic crop, which produces a natural fibre, provides edible protein for livestock feed and is a source of oil and biofuel. Today, the most extensively cultivated cotton species are the tetraploid *G. hirsutum and G. barbadense.* Recently, whole-genome sequencing analyses of diploid (*G*. *raimondii*; and *G*. *arboreum*) and tetraploid cotton species (*G*. *hirsutum* and *G. barbadense*) have provided valuable reference genomes for cotton, and a number of shotgun sequencing efforts have increased our understanding of the variation in these cotton genomes [26–32]. Upland cotton is a moderate salt-tolerant plant, with a salinity threshold of 7.7 dS m^− 1^ [33, 34]. Studies on the tolerance of cotton to salt stresses have shown that a saline environment affects cotton growth and development and influences its biological and metabolic pathways. In particular, the growth of seedlings is severely reduced under a high-salinity environment. Salt stress affects photosynthesis and respiration, flowering, fibre quality and ion uptake in cotton, influencing both biological and metabolic pathways [35, 36]. A study of lncRNAs in the fibre development of cotton identified 30,550 lincRNAs and 4718 lncNATs using RNA-seq, and these lncRNAs are thought to regulate the expression of other genes through multiple RNA-mediated mechanisms [37]. However, reports on lncRNAs involved in salt-responsive regulation in *G. hirsutum* are lacking.

## Results

### High-throughput sequencing

In order to develop a comprehensive catalogue of lncRNAs in upland cotton under salt stress, we performed whole-transcriptome strand-specific RNA sequencing for three-leaf stage cotton seedlings treated with salt stress (S_NaCl) and control (S_CK), including biological replicates(S_NaCl1,S_NaCl2 and S_CK1,S_CK2). Sequencing was done on the Illumina HiSeq 4000 platform, and 250 bp paired-end reads were generated. We obtained more than 40,000,000 raw sequence reads by RNA-seq. From the raw reads, we identified more than 98% that were clean reads (Table 1). To estimate the quality of the RNA-seq data, we used Fast QC with a phred-like algorithm to calculate the quality score (Q) of each base pair in the reads. The results were a mean Q-value of 40, showing that the RNA-seq data were highly reliable (Additional file 1: Mean sample quality).

**Table 1:** RNA-seq data for four samples

Summary	S_CK		S_NaCl	
	S_CK1	S_CK2	S_NaCl1	S_NaCl2
Raw Reads	43,474,829	46,025,954	43,284,887	47,240,877
Clean Reads	43,474,829	45,678,120	42,839,455	46,971,811
Clean Reads Rate (%)	100%	99.24%	98.97%	99.43%
Unique mRNAs	58,471	58,484	58,805	59,080
Unique lncRNAs	1520	1635	1549	1635

**Table 2:** Putative targets and target mimics of lincRNAs for miRNA

miRNA_Acc.	Target_Acc.	Expectation	UPE	miRNA_aligned_fragment	Target_aligned_fragment	Inhibition
ssl-miR395	lnc_1045	3	14.736	GGGAAAUGUUUGGGGAAACU	AGUUUCCCAAAAGAUUUUCU	Cleavage
stu-miR166d-5p	lnc_174	3	15.884	AGAAUGUCGUCUGGUUCGAG	UUUGAACUGGGUGACAUUCU	Cleavage
bra-miR9555a-5p	lnc_175	3	13.847	UUCUAAGCUUUACGGGAAAC	GUUUUUUGUGAGGUUUAGAA	Cleavage
cpa-miR8142	lnc_361	3	15.344	UGAGGUAAGUAGACAGUAAAGGUU	GAUUUUUGCUGUUGAUUUAUCUCA	Translation
gra-miR7504d	lnc_361	2.5	5.165	AGGAAAAAAAAUCUGAUUUGU	GCAAAUCAGUUUCUUUUUCUU	Translation
gra-miR8766	lnc_361	3	7.582	UUAUUUUGGAAUUAGAAAAGUCGU	GCAAUUUUUUUAAUUC-AAAAUAA	Cleavage
cre-miR1173	lnc_376	3	14.984	AUGGUUGCAAUAGAAAUCAU	AUGGUUUGUAUUUUAACCAU	Cleavage
osa-miR1881	lnc_464	3	16.025	AAUGUUAUUGUAGC-GUGGUGGUG	UGCCACCAUUGCUGCAAUAACAUU	Cleavage
bra-miR9565-3p	lnc_464	3	11.927	CUGAAGCUAGUGAAAGAGAGA	UCUCUCUUUCUCUCGUUUUAG	Translation
gra-miR7502b	lnc_612	3	13.131	UUGUUAAAAGUUUCAUCCAU	AUGGGUGAAAUUUUUGAGAA	Cleavage
gra-miR7502c	lnc_612	3	13.131	UUGUUAAAAGUUUCAUCCAU	AUGGGUGAAAUUUUUGAGAA	Cleavage
ath-miR417	lnc_699	3	10.255	GAAGGUAGUGAAUUUGUUCG	UGAACAGAUUCAUUGCUUUU	Cleavage
gma-miR5376	lnc_699	2.5	9.125	UGAAGAUUUGAAGAAUUUGG	CAAAAUUCUUCUAAUUUUCA	Translation
bdi-miR5200c	lnc_723	3	13.74	UGUAGAUACUCUCUAAGGCU	AGUCUUAUAUAGUAUUUACA	Translation
ath-miR5656	lnc_801	3	15.674	ACUGAAGUAGAGAUUGGGUUU	AAAGACAAUUUCUACUUCGGU	Cleavage
mtr-miR5747	lnc_801	3	11.23	AAAAGAAUACUCAUACAUAACA	UAUUAAGUAUGAGUAUUUGUUU	Cleavage
gra-miR7504d	lnc_828	2.5	14.203	AGGAAAAAAAAUCUGAUUUGUC	GCCAAAUGAGAUUCUUUUUCUU	Translation
tae-miR5049-3p	lnc_828	3	13.657	AAUAUGGAUCGGAGGGAGUA	UGCUCCCUUCAGUUCAUAUU	Translation
mtr-miR5285a	lnc_883	3	15.033	UGGGACUUUGGGUAGAAUUAGGC	GUUUAAUUCUGCCCUAAGUCUCU	Translation
mtr-miR5285b	lnc_883	3	15.033	UGGGACUUUGGGUAGAAUUAGGC	GUUUAAUUCUGCCCUAAGUCUCU	Translation
mtr-miR5285c	lnc_883	3	15.033	UGGGACUUUGGGUAGAAUUAGGC	GUUUAAUUCUGCCCUAAGUCUCU	Translation
miRNA_Acc.	Target_Acc.	Expectation	UPE	miRNA_aligned_fragment	Target_aligned_fragment	Inhibition
bcy-miR529	lnc_886	3	15.175	GAAGAAGAGAGAUGGUAGAG	UUCUCUCAUUUCUUUUCUUC	Cleavage
ath-miR865-5p	lnc_894	3	12.672	AUGAAUUUGGAUCUAAUUGAG	UUCAAUUAGCUCUAAGUUCAA	Cleavage
gma-miR4393a	lnc_894	3	6.465	UGAGAAAAGGACGGCAGAAAAG	CCUUUUUGCUGUCCAUUUCUCU	Cleavage
gra-miR8693	lnc_955	3	9.809	AGGAUGAAAAUAUUGAUGUAG	UUGUAUUAAUUUUUUCAUCUU	Translation
gma-miR1520d	lnc_973	3	13.997	AUCAGAACAUGACACGUGAC	GUUGCGUGUUAUGUUCUGCU	Cleavage
bdi-miR399a	lnc_973	3	13.409	UGCCAAAGGAGAAUUACCCUG	CAGGUUAGUUUUCCUUUGGCU	Cleavage
gma-miR1520l	lnc_973	3	13.635	AAUCAGAACAUGACACGUGAU	GUUGCGUGUUAUGUUCUGCUU	Cleavage
gma-miR4994-5p	lnc_973	3	12.834	GGUUAGCUCAAGGAUCUCAC	AUGAGUUCCUUGAGCUAAUU	Cleavage
ghr-156e	lnc_253	4.5	8.055	UGACAGAAGAGAGUGAGCAC	GUUCUGCUUCUCUUUUGUUA	Cleavage

### Identification and characterization of lincRNAs in cotton

All RNA-seq datasets were mapped to the genome of *G. hirsutum* using TopHat in order to reconstruct the cotton transcriptome. According to the percent of reads mapped to genomic regions, we found approximately 53% clean reads distributed among exonic regions, 41% distributed among intergenic regions and 5.6% distributed across intronic regions in S_CK1 (Additional file 2: Percent of reads mapped to genome regions).

Next, the transcripts were assembled and annotated using Cufflinks. We identified 58,471, 58,484, 58,805 and 59,080 unique mRNAs from the four cDNA libraries (S_CK1, S_CK2, S_NaCl1, S_NaCl2), respectively (Table 1). The remaining reads were filtered according to length and coding potential such that transcripts with length < 200 bp were removed and transcripts with a coding potential > 0 were excluded.

**Fig. 1:**
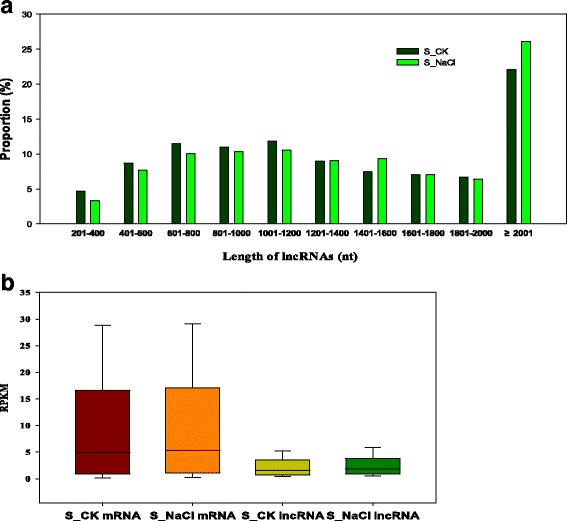
Characteristics of lncRNAs in *Gossypium hirsutum*. **a** Length distribution of lncRNAs. **b** Expression level of lncRNAs and mRNAs in samples

Finally we obtained 1520, 1635, 1549, and 1635 unique lncRNAs from the four samples (S_CK1, S_CK2, S_NaCl1, S_NaCl2), respectively (Table 1). In total we identified 1117 unique lncRNAs in this study. We mapped these lncRNAs to the 13 chromosomes of the *G*. *raimondii* genome and found that they were distributed across these chromosomes without a preference of location in either the controls or treated samples. According to the location of these lncRNAs in the cotton genome, we identified 1117 intergenic lncRNAs (lincRNAs). We then assessed the length and expression level of the lincRNA transcripts. The median length of these lincRNAs was 1200 nucleotides (nt), and most were shorter than 2000 nt (Fig. 1a). We then estimated the expression level of each transcript using reads per kilobase of exon per million fragments mapped (RPKM) and found that the lincRNAs in the control and salt treatment groups were expressed at similar levels (Fig. 1b).

However, the overall expression level of lincRNAs was lower than that of mRNAs (Fig. 1b), consistent with previous studies in *G. barbadense*, humans and *Medicago truncatula* [37–39].

**Fig. 2:**
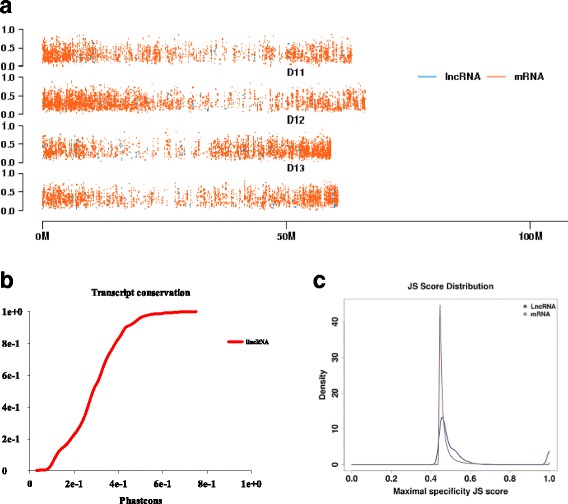
Conservation of *Gossypium hirsutum* lincRNAs. **a**, The conservation score (consScore) of each nucleotide in the *G. hirsutum* genome; (**b**), The level of conservation of the lincRNAs. The cumulative distributions of mean phastCons scores derived from the *G. hirsutum* and Arabidopisis whole-genome alignments are shown; (**c**), The maximal Jensen-Shannon (JS) specificity score distributions for all lincRNAs and mRNAs

Next, we aligned the genomes of *G. hirsutum* [29] and *Arabidopsis* [40] using MultiZ [41] to verify the conservation score (consScore) of each nt using phastCons [42]. We then extracted the consScore of each lincRNA (Additional files 3, 4). Like lincRNAs from rice, we found that the *G. hirsutum* lincRNAs were less conserved than mRNAs (Fig. 2a, b). The lower the conservation level, the lower the conservation score.

We estimated the degree of differential expression between the controls and salt stress samples of the lincRNAs and mRNAs based on the Jensen-Shannon score [39] and found that lincRNAs tended to be more differentially expressed than mRNAs (Fig. 2c). Meanwhile, a strikingly different expression pattern of lincRNAs was found in *Arabidopsis* [19], rice [42], *M. truncatula* [43] and various animals [39], suggesting that these characteristics are conserved for lincRNAs.

### Expression of *G. hirsutum* lincRNAs under salt stress

**Fig. 3:**
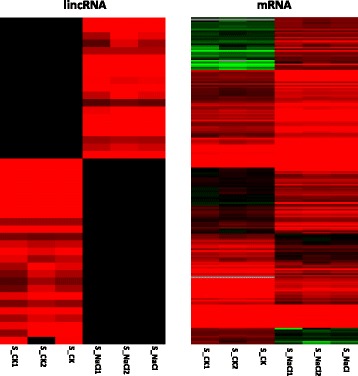
The abundance of specifically expressed lincRNAs (RPKM) and protein-coding genes across 4 samples. The rows and columns were ordered according to Cluster3.0. **a**, 44 specifically expressed lincRNAs.; (**b**), 262 salt-responsive transcription factor protein coding genes and salt tolerance-related genes. Unigene expression values are scaled ranging from + 3 (red) to − 3 (green); S_CK is the average of S_CK1 and S_CK2; S_NaCl1 is the average of S_NaCl1and S_NaCl2. Red represents up-regulated protein coding genes, green represents down-regulated protein coding genes and black indicates no expression of the unigene in the sample. And more details were shown in Additional file 5

**Fig. 4:**
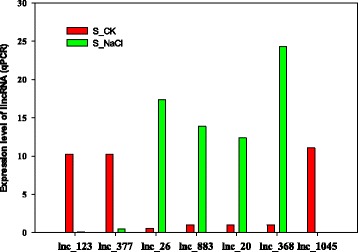
Differential expression analysis of 7 lincRNAs under salt stress. RT-qPCR was performed for 11 randomly selected specific expression lincRNAs from the 44 most specifically expressed candidate lincRNAs under salt stress (Additional file 5 expression level of lincRNAs (RNA-seq). Another four lincRNAs are shown in Additional file 5. UBQ7 expression level was used as the reference gene

To identify salt-responsive linRNAs of *G. hirsutum*, the normalized expression (RPKM) of the lincRNAs was compared between the control and salt treatment groups. We clustered the differentially expressed lincRNAs on the basis of their expression patterns using Cluster3.0. The highly specific expression pattern observed for lincRNAs allowed us to cluster them into two categories, (i) those that were specifically expressed in the controls (for example, lnc_1045, lnc_123), and (ii) those that were specifically expressed under salt-treatment (for example, lnc_26, lnc_388) (Fig. 3a), suggesting that these lincRNAs may function in response to salt stress. We next compared the levels of the salt-responsive protein coding genes in the control samples (S_CK1, S_CK2) and the salt treatment samples (S_NaCl1, S_NaCl2) from the two clusters using hierarchical cluster analysis (Fig. 3b). The log2 ratio values of the salt responsive genes were used for hierarchical cluster analysis with Cluster3.0. Details of the annotations of all differentially expressed protein coding genes shown on the right are provided in Additional file 4. To confirm their expression patterns, we randomly selected 11 lincRNAs and quantified them using real-time quantitative PCR (RT-qPCR), in Fig. 4 and Additional file 5. In Fig. 3a and Additional file 5, the expression patterns of specifically expressed lincRNAs in the sequencing and RT-qPCR results were mostly consistent, although the relative expression levels of all lincRNAs measured by RNA-Seq were greater than those by RT-qPCR. Consequently, four lincRNAs were identified as up-regulated under salt treatment, while two lincRNAs were down-regulated. Additionally, in Fig. 3b and Additional file 6, we found that several salt tolerance-related protein genes were co-expressed with the lincRNAs, for example Gh_A05G3489, Gh_A01G0321, Gh_A01G0639 and Gh_A11G0366.

### Functional analysis of differentially expressed lincRNAs under salt stress

It has been shown that a number of lncRNAs can regulate the expression of genes in close proximity (*cis*-acting) or at a distance (*trans*-acting). Previous studies in animals and plants showed that lncRNAs are preferentially located in close proximity to protein-coding genes that they regulate [43–45].

**Fig. 5:**
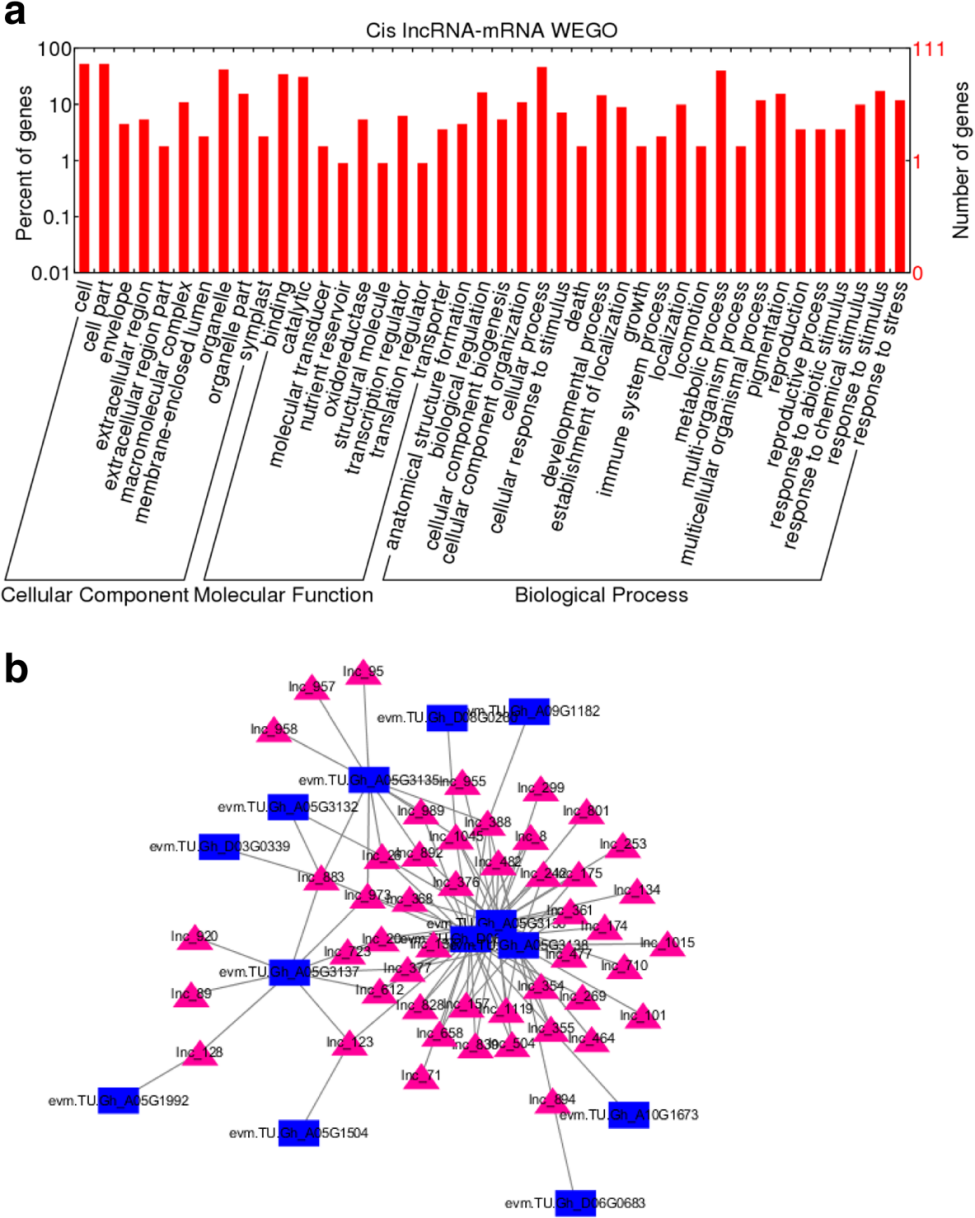
Functional analysis of differentially expressed lincRNAs in salt stress. **a**, Gene Ontology enrichment of co-expressed protein-coding genes with the differentially expressed lincRNAs; (**b**), Representatives of predicted interaction networks among lincRNAs and protein-coding RNAs. The triangular and square nodes represent lincRNAs and protein-coding genes, respectively. Details of interaction networks among lincRNAs and protein-coding RNAs are shown in Additional file 6

To analyse the potential functions of the identified lincRNAs, we selected protein-coding genes that were co-expressed and were spaced less than 20 kb away from the differentially expressed lincRNAs. We analysed the gene ontology (GO) enrichment of these protein-coding genes and found that these protein-coding genes were mainly enriched in stress-related categories, such as “response to stress”, “response to chemical stress”, “biological regulation”, “binging”, “oxidoreductase” and “transcription regulator” (Fig. 5a). In addition, we detected significant enrichment that on a mean *P*-value < 0.05. For example we found GO-term enrichments for biological processes (GO:0006722, triterpenoid metabolic process; GO:0000027, ribosomal large subunit assembly; and GO:0010426, DNA methylation on cytosine within a CHH sequence), cellular component (for example, GO:0016160, amylase activity; GO:0016671, oxidoreductase activity; and GO:0042300, beta-amyrin synthase activity) and molecular function (GO:0010006, Toc complex; GO:0005871, kinesin complex; and GO:0009317, acetyl-CoA carboxylase complex) (Fig. 5b).

These results suggest that the differentially expressed lincRNAs in salt stress may regulate protein-coding genes involved in several important biological processes, such as carbohydrate metabolism, detoxification, energy synthesis, transcription, chromatin modification and post-transcriptional regulation in response to salt stress. lncRNAs can directly regulate the polymerase II transcription machinery in many ways. To illuminate the function of differentially expressed lincRNAs in salt stress and the relationship between lincRNAs and mRNAs which were co-expressed and fall less than 20 kb away from differentially expressed lincRNAs, putative interactive networks were established using Cytoscape (Additional file 7 Network and Fig. 5b). Genes at network nodes were sorted into three groups. First, seven protein-coding genes were involved in oxidation/reduction reactions. These included, for example, the genes encoding peroxidase and thioredoxin. Thioredoxins are small enzymes that participate in redox reactions via the reversible oxidation of an active center disulfide bond. Second, four protein-coding genes were involved in the transport of anions. For example, three genes encoded proteins that are involved in the transport of anions across the cytoplasmic membrane during salt response. Finally, five protein-coding genes were involved in transcription, including Leucine rich repeat, eukaryotic translation initiation factor 5 and RNA recognition motif (Fig. 5b and Addition file 8).

**Fig. 6:**
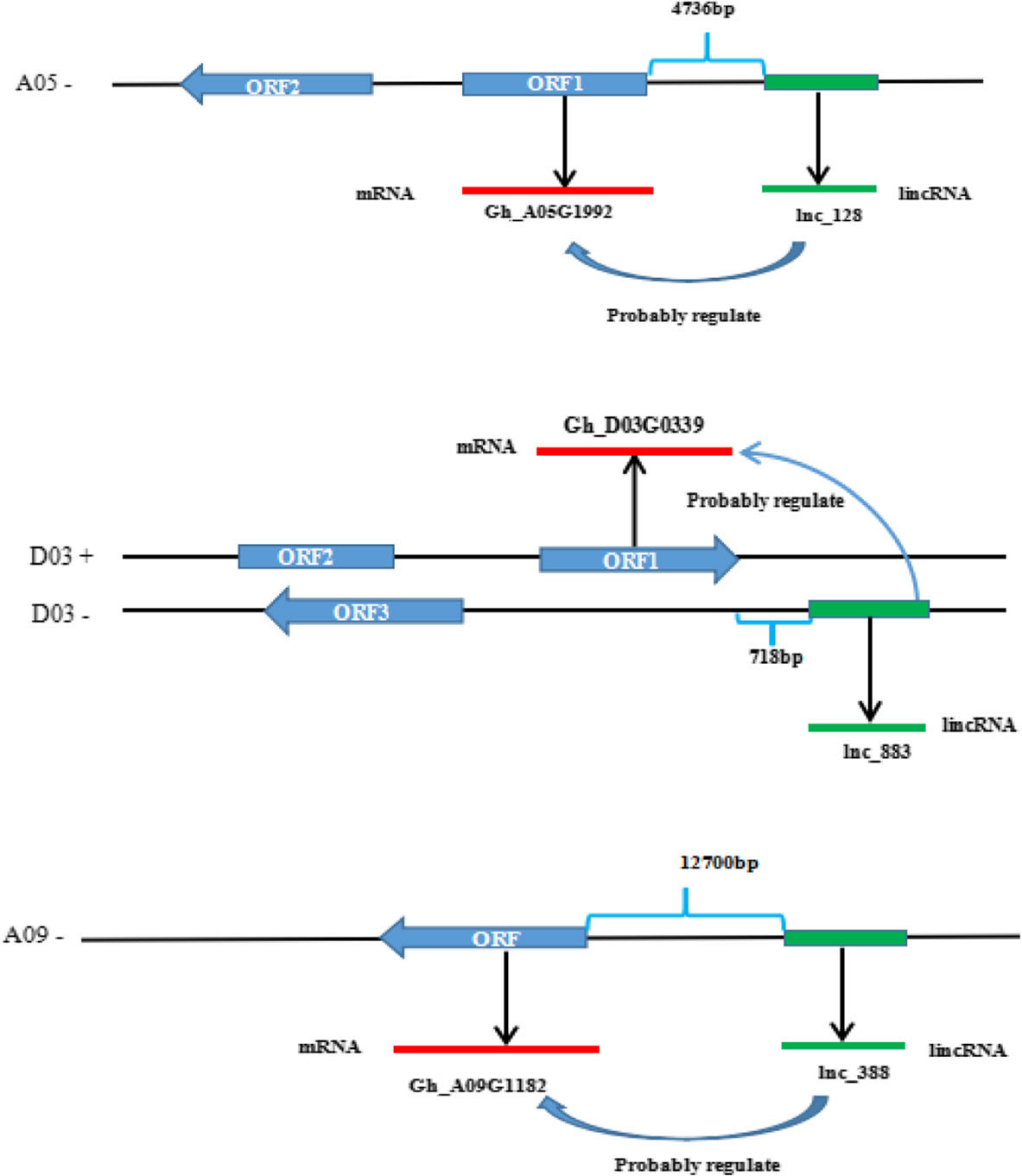
Structure of lincRNAs and their putative target genes

**Fig. 7:**
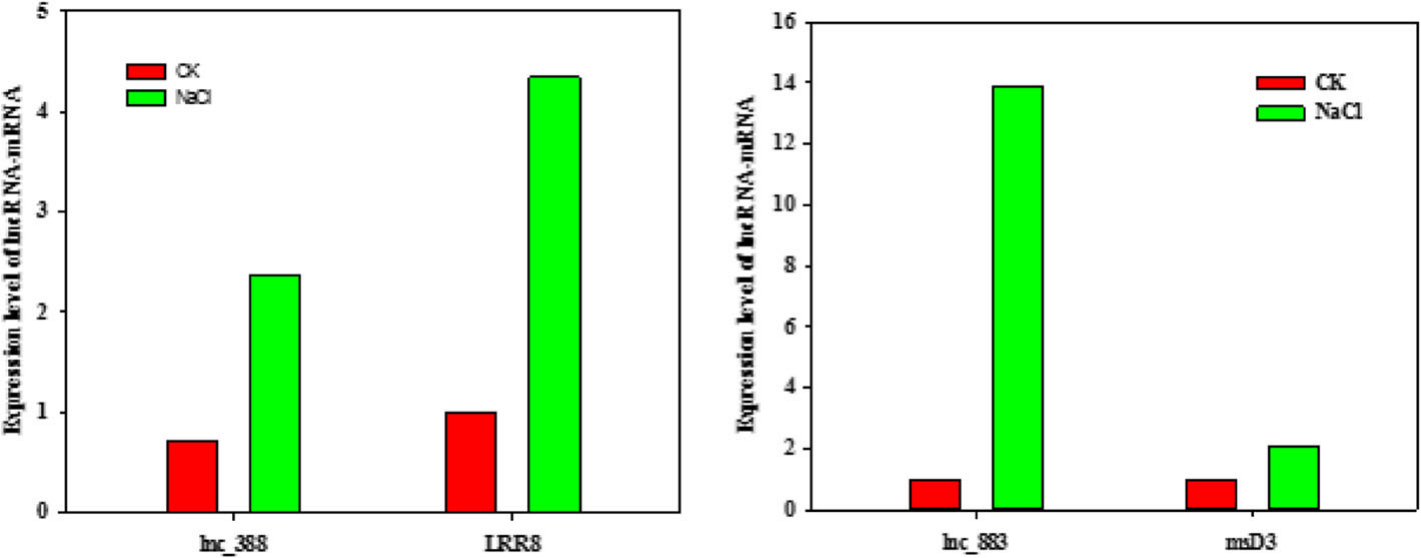
The analysis of RT-qPCR with lncRNA and their putative target genes. LRR8: Leucine rich repeat8 (Gh_A09G1182); msD3: MS_channel (Gh_D03G0339)

Interestingly, we found that 13 lincRNAs spaced less than 20 kb away from their putative *cis-acting* targets may regulate neighboring protein coding genes by *cis-*acting in Additional file 8. Of which,lnc_128 was spaced 4736 bp with Gh_A05G1992 coding eIF-5_eIF-2B, especially lnc_883, located approximately 718 bp upstream of the coding sequence of Gh_D03G0339 (Fig. 6). Another lnc_RNA lnc_388 localized at -strand A09, had a 12.7 kb distance with LRR8 (Gh_A09G1182). To confirm the relationship of lincRNA and neighboring protein coding genes, we selected two lincRNAs we were interested in. Using real-time quantitative PCR (RT-qPCR) lincRNA and their putative *cis-acting* targets were quantified in Fig. 7. We found lnc_388 and putative *cis-acting* targets LRR8 (Gh_A09G1182) were co-expressed and dramatically up-regulated in salt stress. Meawhile the expression of lnc_883 and msD3(Gh_D03G0339) was up-regulated (Fig. 7). These results suggest that lnc_883 may participate in regulating tolerance to salt stress by modulating the expression of Gh_D03G0339 MS_channel. And lnc_388 may be involve in salt tolerance by adjusting the expression of Leucine rich repeat8 (Gh_A09G1182).

### Cotton lincRNAs as endogenous target mimics for miRNAs

**Fig. 8:**
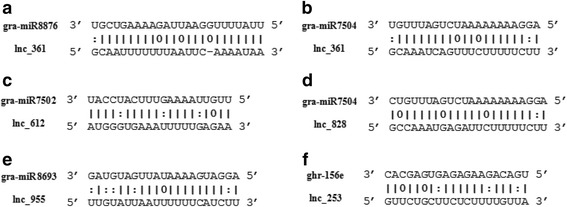
Putative targets and target mimics of lincRNAs for miRNA. lincRNAs as miRNA targets and target mimics are shown in **a b**; **c**; **d**; **e**: **f**

In plants, an important function of lincRNAs is target mimicry, and the miRNA-lincRNA relationship was recently discovered in *Arabidopsis* [46–48]. The first target mimic identified, *IPS1*, was discovered in *Arabidopsis*, in which it modulates the activity of miR-399 by a complementary mechanism [46]. It is possible that functional miRNA target mimics consist mainly of lncRNAs. In this study, the target mimics of these lincRNAs were predicted by psRNAtarget (http://plantgrn. noble.org/psRNATarget) [48, 49]. In total, six and 30 target mimics were identified in *Gossypium* spp. and in other plants, respectively (Table 2: Analysis of target mimicis of lncRNAs for miRNAs, and Fig. 8). Five miRNAs (gra-miR7502, gra-miR8876, gra-miR8693, gra-miR7504d, and ghr-156e) with various functions (i.e. target and target mimicry) were identified (Fig. 8). Lnc_361 targets gra-miR7504 and gra-miR8876. For gra-miR7504, the function appears to be more complex, as it is targeted by two lncRNAs (lnc_361 and lnc_828). And lnc_253 was predicted to target ghr-156e. Therefore, lnc_361, lnc_828 and lnc_253 may regulate their opposite miRNA as target mimics and inhibit its function.

In addition, lnc_973 was identified as a target mimic for bdi-miR399a. By sequence alignment, we found that ghr-miR399a and bdi-miR399a differ at the 16th basepair, which is an A in bdi-miR399a and a G in ghr-miR399a (Additional file 9). Thus, lnc_973 may regulate the expression of ghr-miR399 as a target mimic under salt stress.

**Fig. 9:**
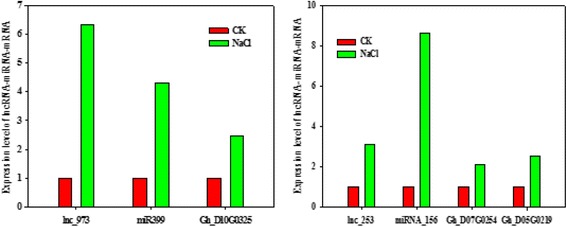
The expression results of lncRNA, miRNA and putative target genes of miRNA. RT-qPCR was performed for lncRNA and miRNA under salt stress, UBQ7 expression level was used as the reference gene

To investigate the mechanism function of lncRNAs (lnc_973, lnc_253), we analysed the expression of lncRNA and corresponding microRNA under salt stress. Interestingly, we found the expression pattern of lnc_973 and corresponding microRNA ghr-miR399a were identical. lnc_973 and ghr-miR399a were up-regulated under salt stress, but the target genes of miRNA Gh_D07G0254 and Gh_D05G0219 appeared up-regulated. Meanwhile lnc_253 and ghr-156e shown the similar result in Fig. 9. These evidences suggested lnc_973 and lnc_253 may regulate the expression of ghr-miR399 and ghr-156e as a target mimic under salt stress. lncRNA can promote the target of miRNA expression by competing miRNA to downregulate the activity of miRNA.

## Discussion

Salt stress induces osmotic and ionic toxicity and oxidative stress disrupts plant homeostasis [50]. Under stress, the sensor systems in plants are triggered by downstream signalling and transcriptional control cascades, which result in extensive changes in cellular gene expression [51, 52]. In response to salt stress, numerous transcription factors are differentially expressed, such as members of the basic leucine zipper (bZIP), basic helix-loop-helix (bHLH), MYB and NAC families, WRKY and ERF, [53], which play critical roles as transcriptional regulators in plant growth and development [54]. Increasing numbers of functional studies on protein-coding genes and small noncoding RNAs have revealed the high level of complexity of eukaryotic transcriptomes, especially considering the extensive abundance of lncRNAs [55]. LncRNAs are a recently discovered type of molecule with important functions in a wide range of biological processes, including developmental regulation and stress response; nevertheless, the detailed mechanisms involved in these biological processes remain largely unknown [56]. In cotton, several studies have identified small ncRNAs and lncRNAs through small RNA sequencing in fibre development, but no data has been presented for lncRNAs under salt stress. The recent publication of genome sequences and the accumulation of RNA-seq data have allowed the genome-wide identification of lncRNAs. In this study, we identified more than 58,000 mRNAs and 1117 unique lncRNAs by analysing more than 40,000,000 raw sequence reads. The number of lncRNAs identified by sequencing were selected based on strict criteria. Although this may have excluded many lncRNAs, these 1117 unique lncRNAs constitute a reliable set of cotton lncRNAs. According to the location of these lncRNAs in the cotton genome, these 1117 unique lncRNAs are lincRNAs.

Moreover, we identified 44 lincRNAs that were differentially expressed under salt stress. The ability of lncRNAs to bind to protein partners endows them with several regulatory abilities. Despite our limited knowledge from relatively few characterized examples, several mechanistic themes of lncRNAs function have emerged, such as functions as decoys, scaffolds and guides [3]. lncRNAs have previously been parsed by whether the guidance occurs in *cis* (on neighbouring genes) or in *trans* (on distantly located genes). The *cis* action presumably occurs in a co-transcriptional manner, leading to the analogy of lncRNAs as tethers [55]. However, recent experiments where ectopically supplied lncRNAs seek out their cognate target sites show that even *cis*-acting lncRNAs have the capacity to act in *trans* [57–59]. Future studies that allow global mapping of lncRNA sites of action may better define the *cis* vs. *trans* nature of lncRNA action. To understand the function of differentially expressed lincRNAs under salt stress, we analysed protein-coding genes that were co-expressed with these lincRNAs. We analysed the GO-term enrichment of these protein-coding genes. We found that these protein-coding genes were mainly enriched in stress-related categories, such as “response to chemical stress”, “biological regulation”, “oxidoreductase” and “transcription regulator” Interestingly, 7 lincRNAs were identified which fall less than 5 kb from their putative *cis* targets These lincRNAs may target protein-coding genes via *cis* regulation.

Currently, there have been few studies investigating the function of plant lncRNA and limited evidence is available to provide detailed information about the functions and regulatory mechanisms of lncRNAs. In 2007, studies in *A. thaliana* identified an endogenous lncRNA, *IPS1*, which can bind to ath-miR399 with a three-nucleotide bulge between the 10th and 11th positions of ath-miR399 at its 5′ end [46]. Such pairing abolished the cleavage effect of ath-miR399 on *IPS1*; thus, *IPS1* serves as a decoy for ath-miR399 to interfere with the binding of ath-miR399 to its other targets protein gene *PHO2*. This type of inhibitory mechanism of miRNA function is termed target mimicry, and *IPS1* a target mimic of miR399. Subsequently, in mammals, the category of target mimicry lncRNAs was renamed to competing endogenous RNAs(ceRNAs) and was shown to be relevant in many processes [50, 56, 60, 61], implying that these molecules might represent a widespread form of gene regulation. lncRNAs can regulate their corresponding miRNA target genes by having miRNA-binding sites and competing for shared miRNAs. In *Arabidopsis*, after the *IPS1*-target mimic miR399 was identified, Wu et al. [62] predicted endogenous target mimics (eTMs) for 20 conserved miRNAs from intergenic or nc gene-originated regions in *Arabidopsis*, rice, *Populus trichocarpa* and maize, and several *Arabidopsis* eTMs have been shown to be functional [48, 56, 63, 64]. We also predicted that specifically-expressed lincRNAs under salt stress act as endogenous target mimics for conserved miRNAs in cotton. Four miRNAs (gra-miR7502, gra-miR8876, gra-miR8693 and gra-miR7504d) with different functions (target and target mimicry) were identified. lnc_973 and lnc_253 were identified as the target mimic for bdi-miR399a (*Brachypodium distachyon)* and ghr-156e. And the result of RT-qPCR shown miRNA399 is not only expressed in phosphate starvation (Bari Rajendra et al., 2006). Moreover, we proved lnc_973 and ghr-miR399a were co-expressed and up-regulated under salt stress. Meanwhile lnc_253 may act as the target mimic for ghr-156e. The importance of lincRNAs in their role as eTMs during plant development and reproduction regulation will continue to emerge in future studies.

## Conclusion

In this study, using RNA sequencing, 1117 unique lncRNAs were identified and 44 lncRNA that were identified as intergenic lncRNAs (lincRNA) differentially expressed under salt stress. Real-time quantitative PCR confirmed that all selected lincRNAs responded to salt stress, which is consistent with RNA-seq. We analysed the gene ontology enrichment of *cis-acting* targets and found that *cis-acting* target protein-coding genes were mainly enriched in stress-related categories. We found lnc_388 likely as regulator of Gh_A09G1182. And lnc_883 may participate in regulating tolerance to salt stress by modulating the expression of Gh_D03G0339 MS_channel. Six miRNAs in *Gossypium* spp. were identified, and lnc_973 and lnc_253 may regulate the expression of ghr-miR399 and ghr-156e as a target mimic under salt stress. In cotton, several studies have identified small noncoding RNAs (ncRNAs) and lncRNAs through small RNA sequencing in fiber development, but no data has been presented for lncRNAs under salt stress. We found these lincRNAs may target protein-coding genes via *cis-*acting regulation. We also discovered that specifically-expressed lincRNAs under salt stress may act as endogenous target mimics for conserved miRNAs in cotton. These findings extend the current view on lincRNAs as ubiquitous regulators under stress conditions.

## Methods

### Plant materials and NaCl treatments

In this study, SN91–11, a salt-tolerant cotton cultivar, was used. This cultivar was obtained by introducing Bluish Dogbane (*Apocynum venetum*) DNA into LM-6, which is a salt-sensitive cotton cultivar, by the pollen tube pathway. The physiological characteristics of SN91–11 have been described in earlier studies [65–67]. Sterilized seeds of SN91–11 were germinated in a mixture of peat and vermiculite at 28 °C. Then, the seedlings were grown under the following conditions: 28 °C/22 °C as day and night temperatures, respectively, under 16 h of light alternating with 8 h of darkness. At the three-leaf stage, the seedlings showing normal growth were randomly divided into two groups; one group was placed into tanks filled with a 250 mM solution of NaCl, and the remaining seedlings were transferred to tanks filled with plain water to serve as controls. After exposure to the two solutions for 24 h, seedlings of the control and treated groups were harvested directly into liquid nitrogen and stored at − 80 °C until used for RNA extraction.

### Construction of lncRNA sequencing library and RNA-sequencing

Total RNA was isolated from each cotton seedling sample using the RNAprep Pure Plant Kit Polysaccharides & Polyphenolics-rich (TIANGEN Biotech, Beijing, China). TRibosomal RNA was removed using the Epicentre Ribo-Zero Gold Kit (Epicentre, USA). Subsequently, sequencing libraries were generated following manufacturer recommendations with varied index labels by the NEBNext Ultra Directional RNA Library Prep Kit for Illumina (NEB, Ipswich, USA). The libraries were sequenced on the Illumina HiSeq 4000 platform, and 150 bp paired-end reads were generated in.

### lncRNA identification

We processed raw data by removing the adaptor-polluted reads, removing the low-quality reads and trimming the reads whose number of N bases accounted for more than 5% (quality score, Q ≥ 30). The reference *G. hirsutum* genome and the annotation files were downloaded from the CottonGen database (http://www.cottongen.org). We built the genome index using Bowtie2 v2.2.3 [68], and clean data was mapped to the *G. hirsutum* genome using TopHat v2.0.12 [69]. TopHat calls Bowtie2 for mapping, which makes it more accurate and fast. TopHat specialized software for transcriptome sequencing reads mapping can identify exon-exon junctions by splitting the mapped reads and mapping them to the reference genome again. According to the characteristics of lncRNA, we adopted seven steps to identify lncRNAs from the transcripts of transcriptome assemblies [55]: (1) transcripts with length < 200 bp and exon count < 2 were removed; (2) every transcript with a coverage of < 3 calculated by Cufflinks were selected; (3) the known protein-coding transcripts were removed; (4) transcripts were removed that were known ncRNAs; (5) transcripts were aligned in the Swiss-Prot and Pfam databases to remove those encoding proteins and protein-coding domains; (6) transcripts were eliminated that did not pass the protein-coding-score test using the Coding Potential Calculator (CPC), Coding-Non-Coding Index (CNCI) and Coding Potential Assessment Tool (CPAT) [45, 70]. A transcript was deemed to be noncoding if its protein potential scored less than 0, which meant that the transcript has no capacity of coding a protein; and (7) transcripts were removed that were detected in only one sample.

### Expression analysis

We estimated the expression of lncRNA and mRNA transcripts using all mapped reads mapping by Cufflinks [71]. First, all RNA-seq datasets were respectively aligned to the cotton genome using TopHat 2.0 [72]. Then, the transcriptome from each dataset was independently assembled using Cufflinks 2.0. All transcriptomes were merged to produce a final transcriptome using Cuffmerge. After the final transcriptome was generated, the abundance of all transcripts was estimated using Cuffdiff according to the final transcriptome, and a BAM file was produced from the TopHat alignment. The remaining transcripts in this study had biological replicates among each group, so we identified differential gene expression analysis using DESeq v1.16 (based on a negative binomial distribution). A *P*-value was assigned to each gene and adjusted by the Benjamini and Hochberg approach for controlling the false discovery rate. Genes with Q ≤ 0.05 and |log2_ratio| ≥ 1 were identified as differentially expressed genes.

### Analysis of lncRNA function

In order to predict the function of salt stress-responsive lncRNAs, the neighbours of lncRNA protein-coding genes were analysed by GO enrichment, and GO terms with Q < 0.05 were considered to be significantly enriched [45]. In accordance with previous investigations, lncRNAs regulate the expression of neighbouring genes though transcriptional activation/repression or epigenetic modification.

### Target mimicry prediction

Targets were predicted by submitting all of the miRNAs (miRBase Release 21, June 2014) and the discovered lincRNAs to psRNATarget (http://plantgrn. noble.org/psRNATarget/) [48, 49], with less than three mismatches and G/U pairs allowed within the lincRNA and miRNA pairing regions. PsRNATarget was used to predict target mimics based on the principles established by Wu et al. [48].

### Quantitative real-time (RT) PCR

To determine the relative transcript levels of selected lncRNAs and protein-coding genes, RT-qPCR was performed with specific primers (Additional file 10: Primer list for gene-specific primers) according to the manufacturer’s instructions for the applied Biosystems 7500 RT-qPCR system (ABI 7500; life technologies Inc., Burlington, ON, Canada) and the SYBR premix ex Taq II system (TaKaRa perfect real time). RNA samples from seedlings of the control and treated plants were collected. Total RNA was isolated from cotton plants using RNAprep Pure Plant kit Polysaccharides & Polyphenolics-Rich (TIANGEN biotech, Beijing, China), and reverse-transcribed using the PrimeScript RT-qPCR kit (TaKaRa, Dalian, China). The RNA concentrations were quantified by a NanoDrop ND-2000 spectrophotometer. The reverse transcription reactions were performed using the primescript RT-qPCR kit (TaKaRa, Dalian, China) according to the supplier’s protocol. The expression profiles of the lncRNAs, miRNA and mRNA were assayed by RT-qPCR. 1 μg of total RNA was used for initiating reverse transcription, and the product was used as template for RT-qPCR using specific primers (primers shown in Additional file 10). Primers were then added to perform PCR. *UBQ7* expression was used as the internal control for RT-qPCR. RT-qPCR was performed as per the manufacturer’s instructions (Takara). Briefly, 2 μl of cDNA template was added to 12.5 μl of SYBR premix ex Taq (Tli RNaseH plus), 0.5 μM concentration of each primer, and ddH_2_O to a final volume of 25 μl. The reactions were amplified for 10 s at 95 °C, followed by 40 cycles of 95 °C for 10 s and 59 °C for 30 s. All reactions were performed in triplicate, and controls (no template and no RT) were included for each gene. The 2^-△△Ct^ method was used to calculate relative gene expression values [73–76].

## Additional files

Additional file 1: Mean quality distribution of samples in this study. (DOCX 52 kb)

Additional file 2: Percent of reads mapped to genome regions in this study. (DOC 111 kb)

Additional file 3: Conservation of *Gossypium hirsutum* lincRNAs. (XLS 5268 kb)

Additional file 4: The conservation score (consScore) of each nucleotide in the *G. hirsutum* genome. (PDF 3617 kb)

Additional file 5: Expression level of lincRNAs. (DOCX 85 kb)

Additional file 6: Table S1. Differentional protein genes (XLSX 282 kb)

Additional file 7: Representatives of predicted interaction networks among lincRNAs and protein-coding RNAs. The triangular and square nodes represent lincRNAs and protein-coding genes. (PDF 41 kb)

Additional file 8: TableS2. Analysis of lncRNA and their putative target (XLS 24 kb)

Additional file 9: Putative targets and target mimics of lincRNAs lnc_973. (XLSX 11 kb)

Additional file 10: Table S3. Primer list for gene-specifi primers. (DOCX 27 kb)

## Abbreviations

ceRNAs: competing endogenous RNAs
CNCI: Coding-Non-Coding Index
CPAT: Coding Potential Assessment Tool
CPC: Coding Potential Calculator
eTMs: endogenous target mimics
lncRNAs: long non-coding RNAs
RPKM: Reads per kilobase of exon per million fragments mapped
RT-qPCR: Real-time quantitative PCR
