# Supplementary figures and images for: Identification of Gossypium hirsutum long non-coding RNAs (lncRNAs) under salt stress
Source: BMC Plant Biol. 2018 Jan 25;18:23. doi: 10.1186/s12870-018-1238-0 (PMC5785843; doi:10.1186/s12870-018-1238-0)

**Mean quality distribution of samples**


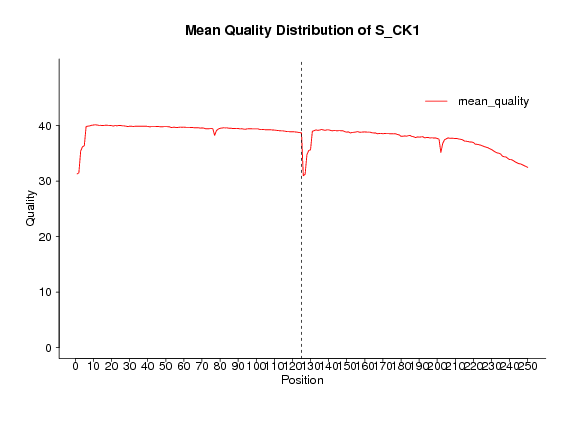


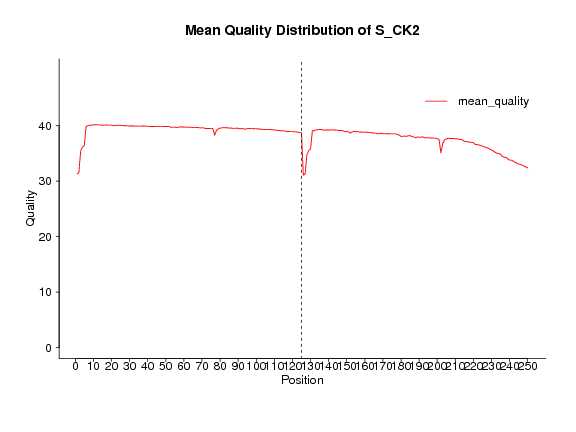


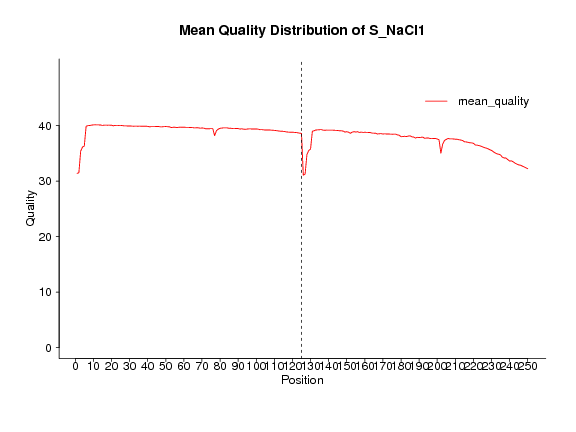


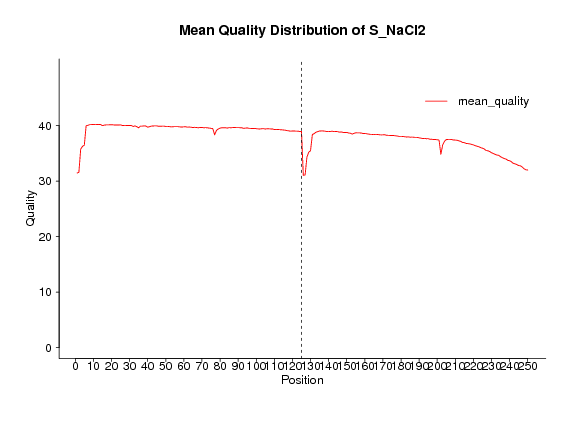

Supplement: Supplementary file 1 — Mean quality distribution of samples in this study. (DOCX 52 kb) [file 12870_2018_1238_MOESM1_ESM.docx]

**Percent of reads mapped to genome** **regions**


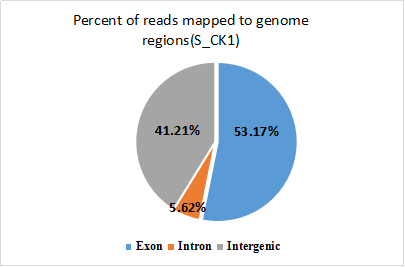

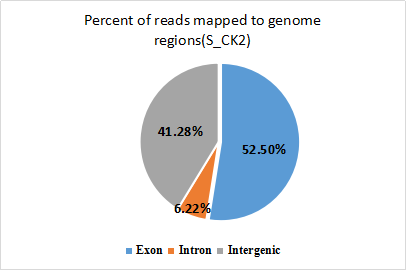

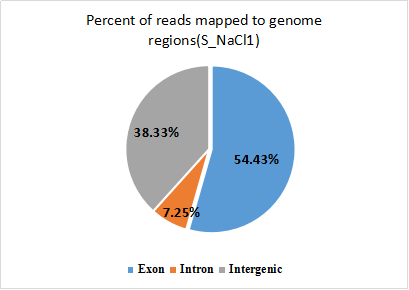

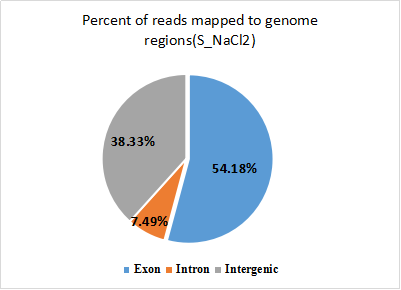

Supplement: Supplementary file 2 — Percent of reads mapped to genome regions in this study. (DOC 111 kb) [file 12870_2018_1238_MOESM2_ESM.doc]

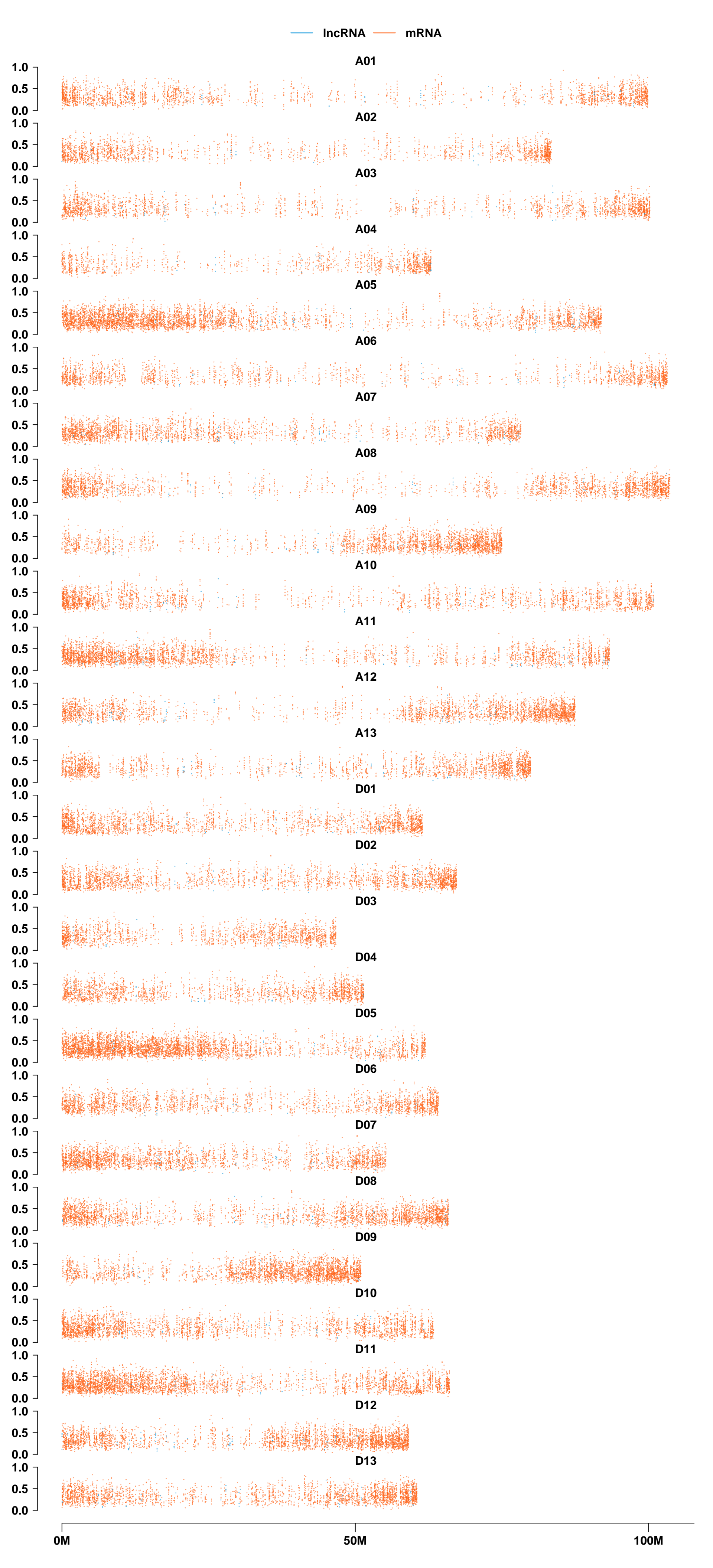

Supplement: Supplementary file 4 — The conservation score (consScore) of each nucleotide in the G. hirsutum genome. (PDF 3617 kb) [file 12870_2018_1238_MOESM4_ESM.pdf]

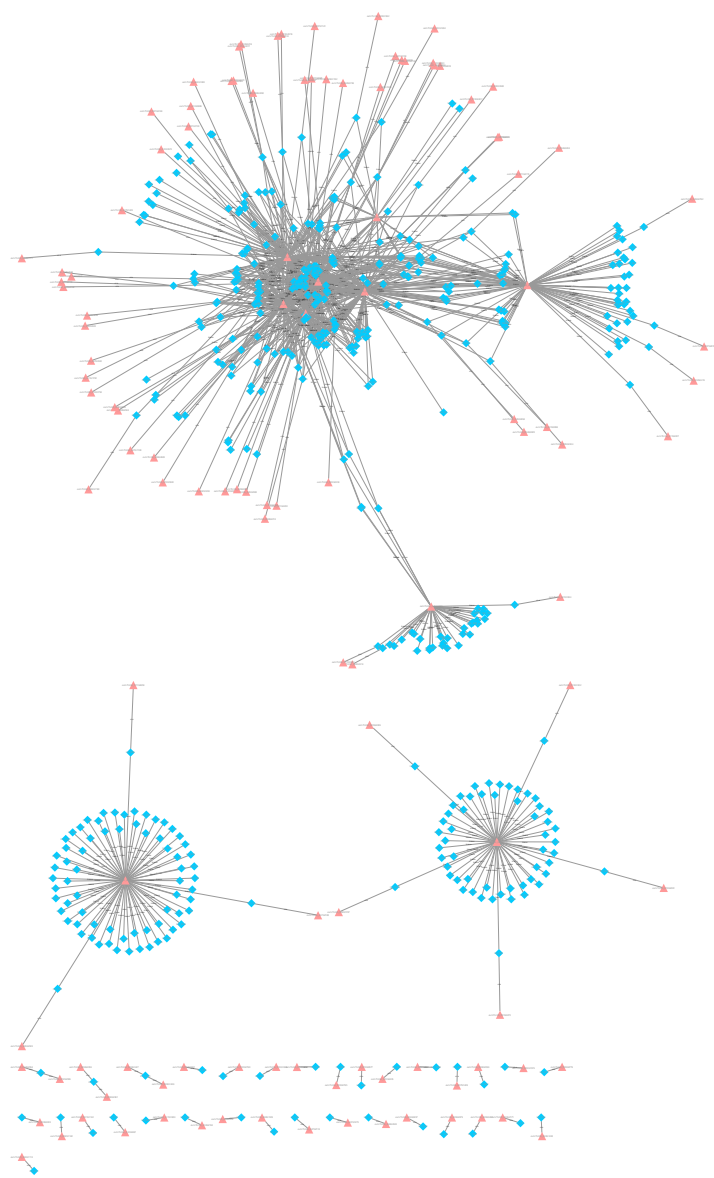

Supplement: Supplementary file 7 — Representatives of predicted interaction networks among lincRNAs and protein-coding RNAs. The triangular and square nodes represent lincRNAs and protein-coding genes. (PDF 41 kb) [file 12870_2018_1238_MOESM7_ESM.pdf]

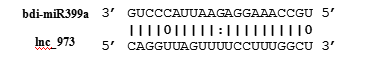

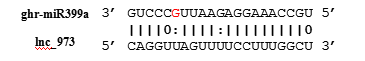


B

A

Fig9. Putative targets and target mimics of lincRNAs lnc_973. lnc_973 as mi399 targets are shown in A; B.

Supplement: Supplementary file 10 — Primer list for gene-specifi primers. (DOCX 27 kb) [file 12870_2018_1238_MOESM10_ESM.docx]
