# Supplementary material for: Identification of Gossypium hirsutum long non-coding RNAs (lncRNAs) under salt stress
Source: BMC Plant Biol. 2018 Jan 25;18:23. doi: 10.1186/s12870-018-1238-0 (PMC5785843; doi:10.1186/s12870-018-1238-0)
Supplement: Supplementary file 5 — Expression level of lincRNAs. (DOCX 85 kb) [file 12870_2018_1238_MOESM5_ESM.docx]

Expression level of lincRNAs RNA-seq and qPCR


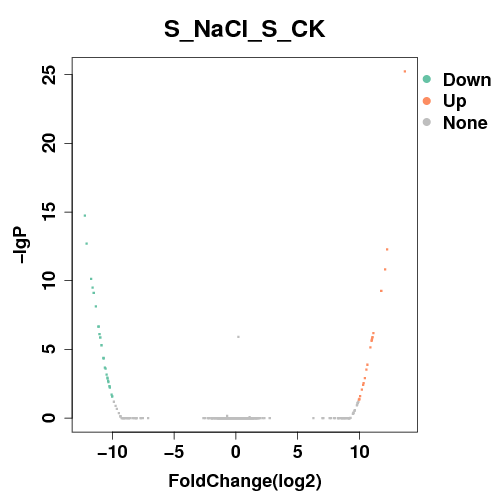


**A**

**1**

**2**

**3**

**4**

**5**

**6**

**B**


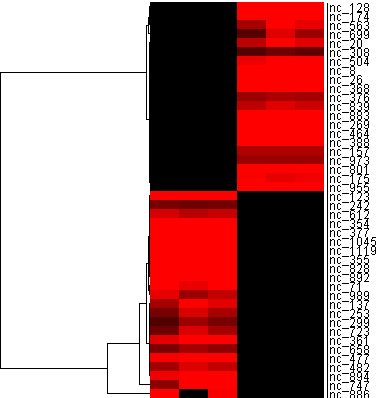


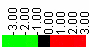


Expression level of lincRNAs RNA-seq under salt stress. A: The differentially expression lncRNA volcano map , the abscissa is expression of fold difference; the ordinate represents significant. B: 3 The abundance of specifically expressed lincRNAs (RPKM). The rows and columns were ordered according to Cluster3.0. Unigene expression values are scaled ranging from +3 (red) to −3 (green). Red represents up-regulated unigenes, green represents down-regulated unigenes and black indicates no expression of the unigene in the sample. 1-2 respectively is S_CK1, S_CK2,3 is the average of S_CK1 and S_CK2; 4-5 respectively is S_NaCl1, S_NaCl2,3 is the average of S_NaCl1and S_NaCl2.

qPCR analysis of 11 randomly selected lincRNA in cotton.
